# Y–Net: Identification of Typical Diseases of Corn Leaves Using a 3D–2D Hybrid CNN Model Combined with a Hyperspectral Image Band Selection Module

**DOI:** 10.3390/s23031494

**Published:** 2023-01-29

**Authors:** Yinjiang Jia, Yaoyao Shi, Jiaqi Luo, Hongmin Sun

**Affiliations:** College of Electrical and Information, Northeast Agricultural University, Harbin 150006, China

**Keywords:** attention mechanism, band selection, convolutional neural network, hyperspectral images, network pruning

## Abstract

Corn diseases are one of the significant constraints to high–quality corn production, and accurate identification of corn diseases is of great importance for precise disease control. Corn anthracnose and brown spot are typical diseases of corn, and the early symptoms of the two diseases are similar, which can be easily misidentified by the naked eye. In this paper, to address the above problems, a three–dimensional–two–dimensional (3D–2D) hybrid convolutional neural network (CNN) model combining a band selection module is proposed based on hyperspectral image data, which combines band selection, attention mechanism, spatial–spectral feature extraction, and classification into a unified optimization process. The model first inputs hyperspectral images to both the band selection module and the attention mechanism module and then sums the outputs of the two modules as inputs to a 3D–2D hybrid CNN, resulting in a Y–shaped architecture named Y–Net. The results show that the spectral bands selected by the band selection module of Y–Net achieve more reliable classification performance than traditional feature selection methods. Y–Net obtained the best classification accuracy compared to support vector machines, one–dimensional (1D) CNNs, and two–dimensional (2D) CNNs. After the network pruned the trained Y–Net, the model size was reduced to one–third of the original size, and the accuracy rate reached 98.34%. The study results can provide new ideas and references for disease identification of corn and other crops.

## 1. Introduction

Corn is an important food crop and industrial raw material, and the stable and healthy development of the corn industry plays an essential role in food security, income generation for farmers, and the national economy [1]. The occurrence of corn diseases can directly affect the yield and quality of corn. Brown spot and anthracnose are the typical diseases of corn, which are very easy to induce and spread under high temperature and high humidity conditions [2]. The two diseases have similar spots but different prevention and treatment methods. To prevent these two diseases, early identification and timely prevention are essential. Currently, growers mainly use visual methods to identify crop diseases [3], which are not only more subjective, less efficient, and have a higher error rate in identifying diseases with high similarity but also easily lead to excessive pesticide inputs, increase agricultural production costs, and cause significant damage to the ecological environment; consequently, they violate the policies advocated and implemented in China, such as “two reductions”, black land protection, etc. Therefore, there is an urgent need to develop a new method to accurately identify anthracnose and brown spot diseases in the early stages of corn growth.

Hyperspectral imaging (HSI) is an effective and non–destructive technique for detecting crop diseases at different spatial scales [4]. Crop infection by diseases leads to changes in physiological characteristics such as moisture, tissue structure, and pigment content, which will alter the crop’s spectral features [5]. As a technique combining spectra and images, HSI can acquire both spectral and spatial features of objects, which gives HSI technology unique advantages in crop disease recognition. Hyperspectral technology has been widely used in the quality detection of agricultural products such as tomatoes [6], rice [7], and leek [8]. However, hyperspectral data contains a large amount of redundant information, and there are certain connections between adjacent bands, which makes it challenging to analyze the data. It is difficult to establish an effective disease recognition model directly. Therefore, feature selection and extraction methods are essential for processing high–dimensional hyperspectral data [9].

In the traditional study of feature selection and extraction from hyperspectral data, Deng X et al. [10] proposed a band selection method based on maximum entropy distance and reverse order selection strategy for identifying citrus yellow dragon disease. Nagasubramanian et al. [11] used genetic algorithms as optimizers and support vector machines as classifiers to determine the best band combinations from 240 bands. Jiang, K. et al. [12] used a successive projection algorithm (SPA) to extract sensitive spectral and textural features associated with mangrove pest and disease information and random forest (RF) to model and visualize leaf features at different pest and disease severity levels. Jiang, J. et al. [13], using principal component analysis (PCA) loading coefficients, selected eight feature bands to identify moldy peanuts. Gao et al. [5] used the least absolute shrinkage and selection operator to choose the characteristic bands for each phenological stage in the dataset and then assessed the sensitivity of the selected characteristic bands based on analysis of variance and linear regression. Finally, least squares support vector machines were used to determine the detectability of effective bands.

While traditional modeling approaches have yielded promising results, many of these models are built based on average spectra without the cooperation of spatial information. This does not take full advantage of the rich information available from HSI in the spectral and spatial domains simultaneously, so there are still limitations when dealing with large datasets [14]. It is widely accepted that the combination of spectral and spatial information can improve the model performance of HSI analysis [15]. For this task, there are usually two approaches. One is to extract texture features and combine them with spectral variables as input to the classifier. The Gray–Level Co–occurrence Matrix (GLCM) is the most commonly used statistical texture feature extraction method. Another strategy for integrating spectral and spatial information is to binaries defective and standard pixels through image processing [16]. For texture features and binarization methods, “manual engineering” involves selecting the region of interest (ROI), principal components, effective wavelengths, texture features, and thresholds for voting or binarization. These choices usually depend on the studied subject and the researcher’s experience. A more objective and automated approach to the work should be adopted.

Deep learning is an emerging tool for learning in–depth features from hyperspectral images. Convolutional neural networks, commonly used as classification networks in deep learning, can be classified as one–dimensional, two–dimensional, or three–dimensional, depending on the size of the filter. In a one–dimensional convolutional neural network (1D–CNN), the spectral vector of hyperspectral image pixels is used as input data to extract spectral attributes and thus classify hyperspectral images [17]. Two–dimensional convolutional neural networks (2D–CNN) can extract features in the spatial dimension of hyperspectral images, but the extraction process loses a significant amount of spectral information. A three–dimensional convolutional neural network (3D–CNN) retains the input spectral information and creates the output volume. In contrast to 2D convolutional neural networks, 3D convolutional neural networks can extract features in both the spatial and spectral dimensions. Preservation of spectral information is essential for hyperspectral images, so 3D convolutional neural networks can obtain the highest classification accuracy, as has been demonstrated in several studies. Ortac et al. [18] proposed 1D, 2D, and 3D neural network architectures for hyperspectral image classification and performed a comparative evaluation. The results show that the 3D–CNN effectively fuses spectral and spatial attributes to achieve the highest performance. Jung et al. [19] developed a 3D–CNN model to classify strawberry grey mold without modifying the input structure of hyperspectral data. They validated its performance advantages by comparing it with a 2D–CNN model.

Although the above study confirms that 3D–CNN classification performance is better than 2D–CNN, 2D–CNN can learn a broader range of spatial features, so combining the two networks can further improve classification performance. Roy et al. [20] combined 2D and 3D convolution advantages by using 3D convolution first, then 2D convolution, and finally connecting the classifier in the designed network. The advantages of 3D convolution are exploited to fully extract spectral–spatial features while avoiding the model complexity resulting from using 3D convolution exclusively. Three convolutional neural networks (lean 2D–CNN, 3D–CNN, 2D–3D–merged CNN) were designed by Chen et al. [21] to implement coffee bean quality detection. The results show that the 2D–3D–merged CNN combines the advantages of 2D and 3D convolutional neural networks, effectively applying spatial and spectral data to achieve the highest detection accuracy.

However, the high correlation between spectral bands in the HSI classification introduces a degree of redundancy. Suppose a 3D–2D CNN model is constructed using the whole band. In that case, it will increase the computational burden and storage requirements, and a large number of spectral bands may lead to “Hughes phenomena” or overfitting of the model. Therefore, some studies reduce the dimensionality of hyperspectral images when developing CNN models so that a small number of hyperspectral image features are used as the input of the model, which makes the classification of hyperspectral images more accurate and faster. Fazari et al. [22] added a trainable linear transform to the beginning of the CNN to reduce the dimensionality of the original hyperspectral image to three dimensions and then combined it with ResNet–101 to detect anthrax in olives. Liu et al. [23] performed dimensionality reduction of spectral features by principal component analysis (PCA) and then fed it into a 3 D–CNN for the classification of hyperspectral images.

Because hyperspectral data contains a large amount of redundant information and some connection between adjacent bands, extracting the essential bands from the raw hyperspectral images is vital. However, dimensionality reduction methods like PCA, independent of the CNN model, are usually inconvenient and less than ideal for handling, making it difficult to achieve global optimality. Therefore, some scholars have taken advantage of the CNN model to extract features by combining the selection of hyperspectral image feature bands with the CNN model. Yuan et al. [24] integrated feature selection, extraction, and classification into an end–to–end trainable network. A point–centered convolutional neural network combined with an embedded feature selection network (PCNN–FS) is proposed to classify hyperspectral images of healthy and aflatoxin–infected moldy peanuts. In the feature selection step, the authors used a 1×1 deep one–dimensional convolution to select the feature bands. Two CNN models, novel convolutional neural network–based feature selector (CNN–FS) and convolutional neural network with attention framework(CNN–ATT), were designed by Zhou et al. [25] CNN–FS is used to extract feature bands; CNN–ATT is a classification model with an attention mechanism. The study first used CNN–FS to acquire feature bands and then trained CNN–ATT with the acquired feature bands. The authors utilized a score vector in CNN–FS to represent the importance of each channel in the target classification. The above two methods split the feature selection block and classification into two steps: training the network with the full band, acquiring the feature band based on this network, and finally retraining a network with the selected feature band cumbersome to split into two steps. Liu et al. [26] developed a two–branch classification model (2B–CNN) and used the weighted sum of the first convolutional layer of a two–dimensional convolutional branch to evaluate the importance of bands; however, the weights of the convolutional layers were obtained by mutual calculation between different channels and were easily disturbed by neighboring bands, so the extracted bands were not representative. Feng et al. [27] proposed a network with independent convolution and hard thresholding that combines band selection, feature extraction, and classification into an end–to–end trainable network. Still, the addition of full–band branches increases the computational effort. Lorenzo et al. [28] combined an attention–based mechanism CNN with anomaly detection to discover the most important bands in HSI. However, only one–dimensional convolution operations are used in the network structure, and only the spectral features of hyperspectral images are considered, while spatial features are ignored.

According to the articles surveyed above, the traditional classifier and feature selection methods are two separate steps that are cumbersome to deal with. Deep learning classifiers outperform traditional classifiers in spectral pattern recognition, and deep learning methods offer more prominent advantages in hyperspectral image feature band selection. This study proposed a 3D–2D hybrid CNN model combined with a band selection module. The band selection module is used to search for spectral channels that are beneficial to the final classification problem, and an auxiliary classifier is combined to update the weights. The 3D–2D hybrid model extracts joint spatial–spectral features from selected spectral bands. This study prepared 200 disease spectral datasets of corn leaves (6264 regions of interest) for the experiment.

The main contributions of this paper are as follows:

(1) The band selection process is incorporated into the training of CNN. It not only overcomes the problem of difficulty in achieving global optimality due to the separation of band selection and classification but also alleviates the time–consuming problem caused by repeated training of the classification network;
(2) The auxiliary classifier of the band selection module not only solves the difficulty that the weights of the band selection module cannot be updated but also optimizes band selection, spatial, spectral feature extraction, and classification;
(3) The constructed 3D–2D hybrid CNN model makes full use of the spectral and spatial features and avoids the model being too complex;
(4) Two similar diseases of corn were identified accurately.

## 2. Materials and Methods

### 2.1. Disease Sample Data Collection

Brown spot and anthracnose are the typical diseases of corn, which are very easy to induce and spread under high temperature and high humidity conditions. Anthracnose spots are the shuttle type, light brown in the center and dark brown all around. Nutritional deficiencies, aphid damage, or improper use of herbicides may also cause anthracnose. Brown spot disease spots are characterized by Water–soaked yellow spots, in which patches often congregate and, in severe cases, cover the entire leaf. 

**Figure 1:**
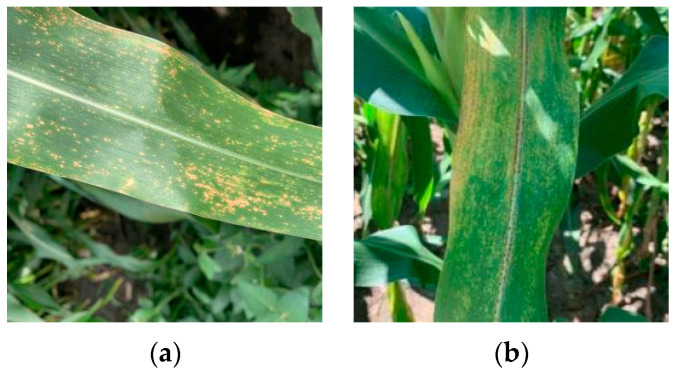
Diseased corn leaves. (**a**) Anthracnose; (**b**) Brown spot.

From 11–15 August 2021, 100 samples of each of the two diseases (anthracnose and brown spot) of corn leaves were collected in Northeast Agricultural University’s experimental field. Both diseases occurred naturally, as shown in Figure 1. The collection time was in the corn filling period; at this stage, corn needs to absorb a lot of nutrients and water to meet the needs of grain development. Poor ventilation and light in the field can easily cause various diseases, including corn anthracnose and brown spot, which are two diseases that are very easy to occur.

The samples were cut off from the root of the leaves with scissors, put into a small, refrigerated box, sealed away from light to reduce the water loss of the leaves, and brought back to the laboratory for hyperspectral data collection.

### 2.2. Hyperspectral Data Acquisition

**Figure 2:**
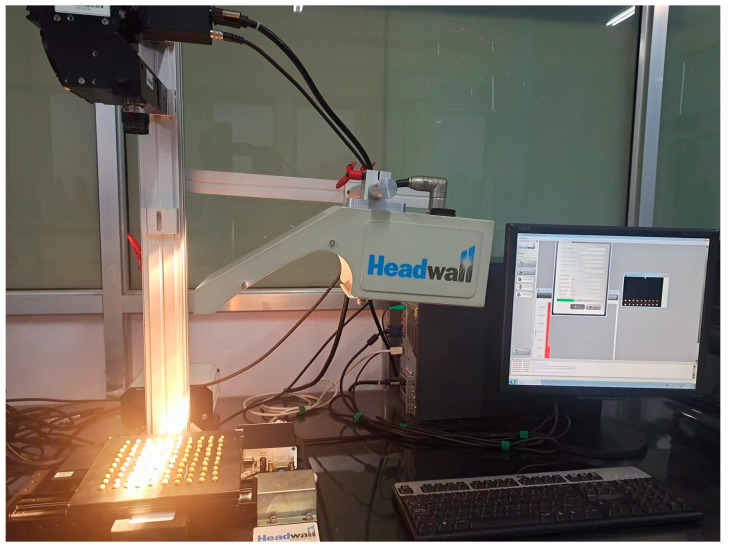
Hyperspectral data acquisition of corn leaves.

This study performed the hyperspectral data acquisition of corn leaves using an HSI system produced by Head Wall, U. S. A. The Head Wall system consists of a hyperspectral camera, a light source (150 W adjustable halogen lamp), a mobile carrier table, a light source box, a collector, and a computer, as shown in Figure 2. The system sensor imaging method is a line array push scan; the spectral range is 400–1000 nm, the spectral resolution is 2.4 nm, and the acquisition interval is 3 nm. For image acquisition, the exposure time was set to 30 ms, and the movement speed of the stage was 5.0 mm/s. The collected corn leaves were laid flat on the color–giving cardboard of the moving platform with the lens vertically downward, 45 cm from the moving platform. Bright current and dark current were calibrated before measurement. The calibration formula was defined based on the following equation:

(1) $$R=\frac{I_{row}-I_{black}}{I_{white}-I_{black}}$$

where $I_{row}$, $I_{black}$, $I_{white}$ represent the experimental spectral reflectance, dark current spectral reflectance, and bright current spectral reflectance, respectively. Hyperspace, the spectral acquisition software of the system, completed leaf hyperspectral data acquisition.

**Figure 3:**
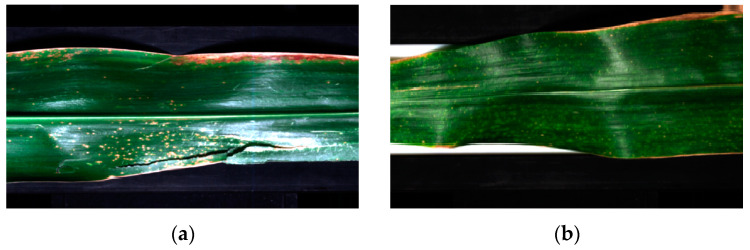
Pre–process results of hyperspectral images of two diseases. (**a**) Anthracnose image after SG processing; (**b**) Brown spot image after SG processing.

To reduce the noise generated by external stray light, sample background, and instrument performance during corn leaf hyperspectral data acquisition, attenuate or eliminate the influence of non–target factors, improve the signal–to–noise ratio, and establish a stable mathematical model, pre–processing of the raw spectral images was required. Savitzky–Golay smoothing (SG) was used in this study. SG eliminates overlapping peaks and provides data baseline correction, reducing noise interference and improving the smoothness of the spectrum, as shown in Figure 3. Next, three or four 30 × 30 rectangular areas were selected as areas of interest in each image and ensured that each area contained disease spots. Three hundred forty–eight areas of interest for anthracnose and 348 for brown spot leaves were obtained as samples, respectively.

For convolutional neural networks, a small data set can easily lead to overfitting. To avoid this, this study divided each 30×30 region of interest into nine 10×10 areas to expand the number of samples. A total of 6264 disease samples were obtained. 

### 2.3. Traditional Feature Selection Methods and Classifiers

#### 2.3.1. Feature Selection Methods

The commonly used feature selection methods Successive Projection Algorithm (SPA) [29] and Partial least squares regression (PLS) were used to compare with the method proposed in this study. SPA is a forward cyclic selection method. The algorithm performs cyclic calculation from the first band, adds the band with the largest projection vector to the feature band set in each calculation, and ensures that each newly added band has the minimum linear correlation with the existing band to minimize the data redundancy. PLS is the orthogonal decomposition of the measured spectral matrix to eliminate information overlap. The selected features are further processed using a classification model. The selected features are further processed using a classification model.

#### 2.3.2. Classifiers

In this study, the feature bands extracted from SPA and PLS were fed into a Support Vector Machine (SVM) for crop disease classification. SVMs improve generalization through structural risk minimization and show outstanding advantages in data processing problems with small samples, non–linearities, and high–dimensional feature spaces [30]. The Radial Basis Function (RBF) was used to establish the SVM classification model, and −32–32 was set as the search range for the penalty parameter c and the kernel function parameter g of the SVM. The initial parameters are randomly generated numbers within the search value range. The Grid–search method is used to determine the best combination of the SVM parameters to maximize the classification accuracy of the modeled samples. The modeling samples were calibrated using the K–Fold cross–validation (Cross–validation) method for the models. Data and analyses for the traditional way were performed in ENVI 5.3 and MATLAB 2016 R2016b.

### 2.4. Y–Net Architecture

**Figure 4:**
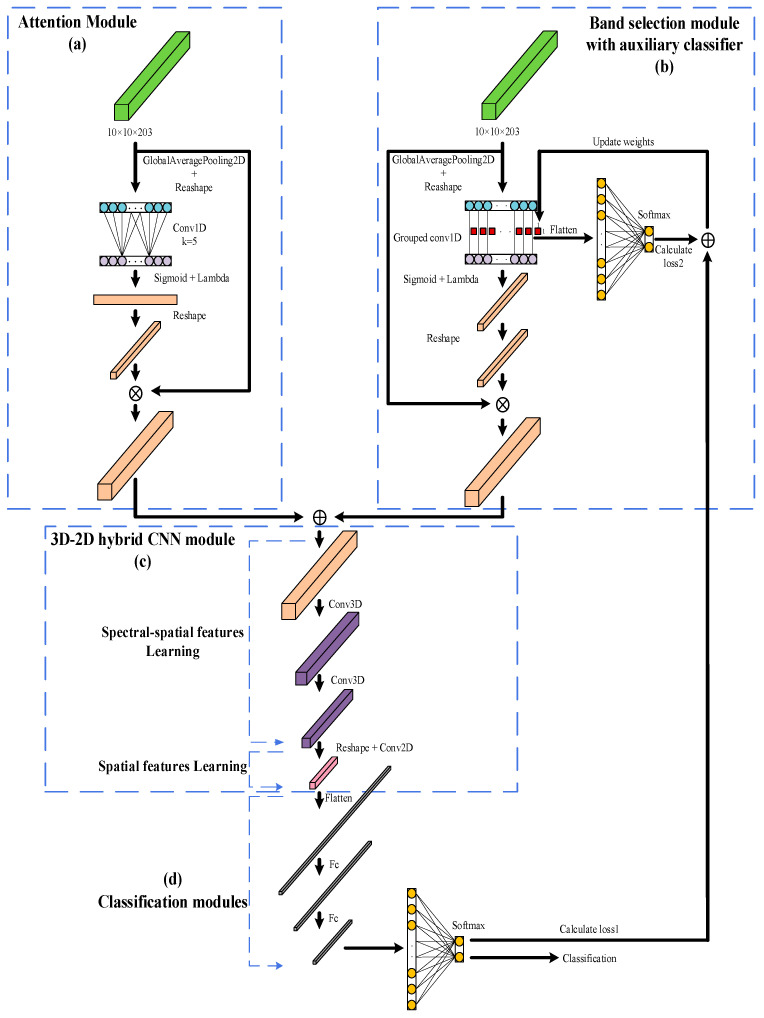
Y–Net architecture. (**a**) Attention module; (**b**) Band selection module with auxiliary classifier; (**c**) 3D–2D hybrid CNN module; (**d**) classification module.

The input to the model in this study was 10×10×203 hyperspectral cube data, where 10×10 was the size of the input data image, 203 was the number of bands, and each data has a label. The architecture of Y–Net is shown in Figure 4. The main structure consists of four parts, namely the attention module (a), the band selection module with auxiliary classifier (b), the 3D–2D hybrid CNN module (c), and the classification module (d). The four modules are annotated with dashed boxes and arrows, and the inputs to both the band selection module and the attention module are hyperspectral image data at 10×10×203. A 1×1 convolution was performed independently for each band of the input hyperspectral image in the band selection module. Then the band selection module weights were updated during training using an auxiliary classifier. The band selection and attention modules’ outputs were then summed and fed into the subsequent layers. To make full use of the spectral and spatial information of the HSI data, a hybrid convolutional feature extraction layer was designed. Finally, the learned spatial and spectral features were fed into the classification layer.

#### 2.4.1. Band Selection Module

HSI is an up–and–coming method for crop disease identification. However, hyperspectral data are mixed with a large amount of redundant information, which makes it more difficult to establish an effective disease classification model. This study uses a CNN model for band selection to solve this problem, retaining only the most representative and information–rich bands in the original hyperspectral images.

The band selection module was constructed by grouping 1×1 one–dimensional convolutions, with the convolution operation performed independently of each band of the input hyperspectral image and updating the weights of the convolutional kernel during the first half of the network training using a variable loss factor and an auxiliary classifier. The weight of the convolution kernel of a one–dimensional convolution layer indicates the importance of the band; the more significant the absolute value of the weight, the more critical the corresponding band is. This is because traditional 1D convolution runs on adjacent regions centered on the target feature channel rather than on each channel individually. This means that the weights of the convolutional layers are obtained by the mutual calculation between different channels, which are easily interfered with by neighboring bands and cannot accurately obtain the importance of the single band. Therefore, the group convolution without bias was selected here. In the group convolution, each channel has a separate convolution kernel running on the channel, which will not be affected by neighboring bands. The band selection module can be expressed as follows:

(2) $$Y_{Band–selection}=f_{A}\left(W_{Con1D}\cdot X\right)$$

(3) $$X=\left[X_{1},\;X_{2},\;X_{3},\;\ldots ,\;X_{n}\right],\;W_{Con1D}=\left[W_{1},\;W_{2},\;W_{3},\;\ldots ,\;W_{n}\right]$$

$Y_{Band–selection}$ denotes the spectral data after weighting; $W_{Con1D}$ is the convolution kernel weight of the 1D convolution layer in the band selection module; X is the input data; n denotes the number of bands. To maintain the fairness of each band channel before training, $W_{Con1D}$ was initialized as $\left[1,\;1,\;1,\;\ldots ,\;1\right]$; that is to say, before training begins, the importance of each band channel is equal. “ · ” represents the multiplication of the elements of two matrices. $f_{A}$ is the activation function; common activation functions are the Sigmoid activation function, Tanh activation function, Relu activation function, etc., where the Sigmoid activation function maps a real number to the range (0, 1). However, the purpose of band selection is to remove some unimportant band channels. During training, the scores of unimportant band channels are usually close to or less than zero. These features also impact subsequent classification, so using the Sigmoid activation function is inappropriate. Similarly, the Tanh activation function compresses the real numbers to the interval −1–1, which is inappropriate. So, the Relu activation function was selected here. The Relu activation function gives an output of 0 when input x<0 and x when x≥0. This activation function allows the network to converge more quickly and does not saturate.

#### 2.4.2. Auxiliary Classifier

To ensure the updating of the weights in the band selection block in the early stage of the model, an auxiliary classifier was added to the band selection. The input of the auxiliary classifier is the output of the one–dimensional convolution layer of the band selection module, namely $W_{Con1D}\cdot X$. Then, the loss function of Y–Net was defined by combining the loss of the final classifier and the auxiliary classifier. The formula is as follows:

(4) $$Loss\left(y,\;\tilde{y}\right)=\left(1-\sigma \right)\cdot Loss_{1}\left(y,\tilde{y}\right)+\sigma \cdot Loss_{2}\left(y,\tilde{y}\right)+\alpha \cdot \lambda \cdot \sum_{j=1}^{n}\left|W_{Con1D}\right|$$

(5) $$Loss_{1}\left(y,\tilde{y}\right)=-\frac{1}{N}\sum_{i=1}^{N}\left\{y_{i}\log\left(\tilde{y_{i}}\right)+\left(1-y_{i}\right)\log\left(1-y_{i}\right)\right\}$$

(6) $$Loss_{2}\left(y,\tilde{y}\right)=-\frac{1}{N}\sum_{i=1}^{N}\left\{y_{i}\log\left(\tilde{y_{i}}\right)+\left(1-y_{i}\right)\log\left(1-y_{i}\right)\right\}$$

(7) $$\sigma =1-\frac{t}{T}$$

where $Loss_{1}\left(y,\tilde{y}\right)$ is the loss of the final classifier and $Loss_{2}\left(y,\tilde{y}\right)$ is the loss of the auxiliary classifier, both using cross–entropy loss. $Loss_{1}\left(y,\tilde{y}\right)$ was used to control the classification accuracy, and $Loss_{2}\left(y,\tilde{y}\right)+\alpha \cdot \lambda \cdot \overset{n}{\underset{j=1}{\sum }}\left|W_{Con1D}\right|$ was used to update the weights of the band selection layer. $\alpha \cdot \lambda \cdot \overset{n}{\underset{j=1}{\sum }}\left|W_{Con1D}\right|$ is the sum of the weights of the band selection module, which can constrain the weight sparsity of the band selection module so that the score of unimportant features is close to zero. σ is the adjustment factor, which controls the loss factor of the final classifier and the auxiliary classifier. Where T is the total number of iterations and t is the current number of iterations in the training process. Due to the presence of σ, at the beginning of the iteration, Y–Net tends to update the weights in the band selection module and learn a basic classification model. As t increases, σ gradually decreases from 1 to 0, and Y–Net tends to train the final classifier to learn a more accurate classification model.

#### 2.4.3. Attention Module

The attention mechanism is one way to achieve adaptive attention in the network, allowing the network to focus on the more critical information for the task among the many input data, reducing the concentration to other details. In general, attention mechanisms can be divided into spatial attention mechanisms, channel attention mechanisms, and a combination of the two. Because this study wanted the network to focus on the importance of the bands of hyperspectral image data, the channel attention mechanism was used [31]. The attention mechanism used in this study was computed by encoding the input in two dense layers. However, capturing the dependencies of all channels was inefficient and unnecessary, so a 1D convolution was added to the features after global average pooling for learning. The specific process can be expressed as follows:

(8) $$Y_{Attention\;Module}=f_{A}\left\{f_{Con1D}\left[f_{Avg}\left(X\right)\right]\right\}\cdot X$$

where “·” denotes the multiplication of matrices, $f_{Avg}$ indicates global average pooling, $f_{Con1D}$ represents a one–dimensional convolution operation, $f_{A}$ is the activation function, and the Sigmoid activation function was used here.

Although both the attention module and the band selection module can generate fraction vectors, they represent different functions. In the trained model, the one–dimensional convolution in the attention module was run on an adjacent region centered on the target feature channel. The convolution operation in the band selection module was run on each channel separately. So, the attention module cannot be used for feature selection, but it can be used effectively to build classifiers and improve classification accuracy.

#### 2.4.4. 3D–2D Hybrid CNN Network Structure

For hyperspectral image data, using 2D–CNN alone can only learn spatial features while ignoring spectral features, thus missing the channel information. Using 3D–CNN alone can lead to very complex models. To exploit the feature learning capability of both 2D and 3D CNNs, the study used a 3D–2D hybrid CNN structure for spatial–spectral feature extraction, which not only made full use of the spectral and spatial features but also avoided overly complex models.

In the 3D–2D hybrid CNN, convolution was performed in the first 3D convolutional layer using 8 convolutional kernels with a step size of 1. In the second 3D convolution layer, 16 convolution kernels with step size 1 were used for convolution to extract spatial and spectral features. Then, a two–dimensional convolution was performed using 64 convolution kernels with a step size of 1 to extract deep features further. In the classification module, 2 fully connected layers (with 256 and 64 neurons, respectively) and a SoftMax layer were used to judge the class of corn diseases. The Batch Normalization and Dropout layers were used after each convolutional and fully connected layer to prevent overfitting problems. The network was trained for 500 epochs using a small batch of size 32.

### 2.5. Experimental Environment

The equipment used in this experiment was a deep learning workstation with the following basic configuration: CPU was Intel^®^ Core™ i9–9900K @3.6GHz ×16 processor, the memory graphics card was RTX 3090 24 Gb, memory and storage spaces were 32 Gb and 2.5 Tb, respectively, graphics driver version number was 455.45.01, CUDA version 11.1 was installed, cuDNN version number was 8005. The operating system was 64–bit Ubuntu 20.04 LTS, the programming language was python 3.7.9, and all network structures were written, trained, and tested in a virtual environment using Keras 2.4.3 under the TensorFlow–GPU framework.

## 3. Results and Discussion

### 3.1. Spectral Characteristics and Principles of Healthy and Diseased Corn Leaves

The spectral characteristics of a healthy green plant depend mainly on its foliage. In the visible spectral band, the spectral properties of plants are mainly influenced by chlorophyll. The absorption valley is due to chlorophyll’s strong absorption of radiant energy in the blue band centered at 450 nm and the red band at 670 nm. The reflectance and transmittance of the leaves are very low, and the absorption between the two valleys is relatively reduced, forming a green reflective peak, referred to as the “green peak”, which is visually expressed as green. When plants grow healthily and are at the growth peak with high chlorophyll content, the “green peak” shifts to the blue light direction, while when plants “lose green” due to harm from diseases and insects or a lack of nutrients, the “green peak” shifts to the red–light direction [32]. The spectral action of green plants in the near–infrared band depends on the cellular structure inside the leaf. The spongy mesophyll tissue of healthy leaves is considered to be a good reflector for any radiation when all the space is filled with water and expanded. The gridded soft reticulate cellular tissue in mesophyll tissue absorbs blue and red light in visible light and reflects green light. When a disease attacks a plant, the water metabolism of the leaf tissue is impeded. After that, as pest damage increases, the plant’s cellular structure is damaged. The content of various pigments is reduced, leading to a reduction in the ability of the leaf to reflect near–infrared radiation. The spectral characteristics show an increase in reflectance in the visible region (400–700 nm) and a decrease in the near–infrared region (720–1100 nm). The near–infrared region is studied with a focus on the “red edge”, which is usually located between 680 nm and 750 nm. The position of the “red edge” moves along the wavelength axis depending on chlorophyll content, biomass, and phenological changes. When chlorophyll content is high, and growth is vigorous, the “red edge” is shifted towards the infrared; when plants are “losing green” due to pests and diseases or pollution or weathering changes, the “red edge” will move towards blue light [33].

**Figure 5:**
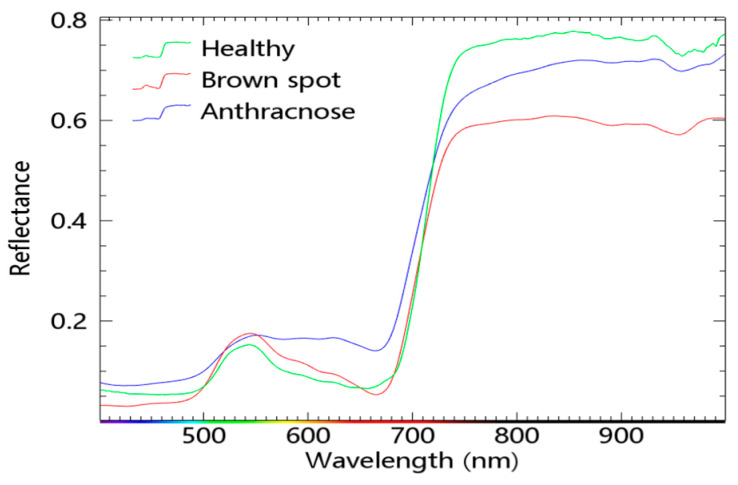
Mean spectral profiles of healthy corn leaves and corn leaves with anthracnose and brown spot.

Figure 5 shows the average spectral curves of healthy maize leaves and maize leaves with anthracnose and brown spot disease. As shown in the above survey, the reflectance in the visible light region (400–700 nm) increases while that in the near–infrared region (720–1100 nm) decreases when crops are affected by diseases. Moreover, the “red edge” moves toward the blue light.

### 3.2. Training Procedure

All images were divided into a training set, a validation set, and a test set according to 5:2:3 and put into the model for training and testing. The Y–Net model uses the Adam optimizer with an initial learning rate of 0.001 and a decay setting of 0.000001 output training by minimizing cross–entropy loss. The loss function is defined in Equation (4). The λ in Equation (4) is initialized to 0.001. Set epoch to 500. The initialization of σ in Equation (7) is 1 and decreases as the number of current iterations increases. In other words, for the first 250 epochs, the network focuses more on training the band selection module and updating the band weights. After 250 epochs, the network focuses on updating the final classifier to improve classification performance. Traditional feature selection methods and classifiers are described in the section “Traditional feature selection methods and classifiers”.

### 3.3. Results of Band Selection

**Figure 6:**
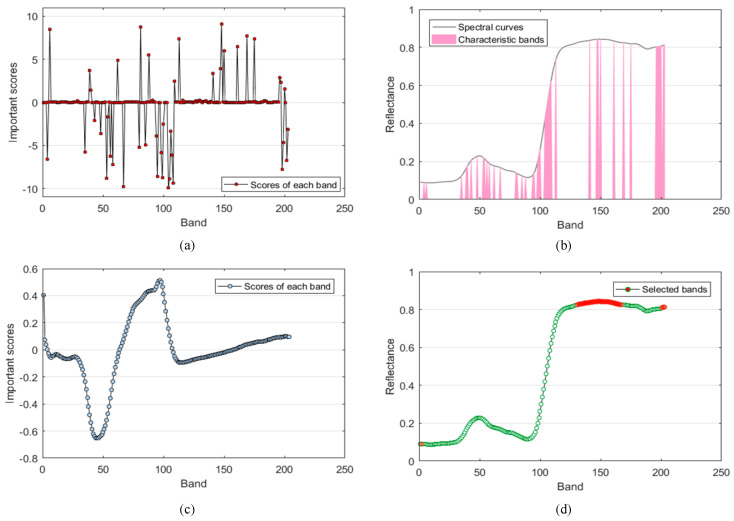
Results of feature band selection. (**a**) The importance score of each feature channel is calculated by the Y–Net band selection module. (**b**) The region of the spectral curve where the feature band extracted by the band selection module is located. (**c**) The magnitude of the regression coefficients of each band by PLS calculations. (**d**) Feature bands by SPA selection.

The feature selection results are shown in Figure 6. The horizontal coordinate of the four figures is the number of bands, totaling 203 bands, ranging from 400–999 nm. Figure 6a shows the characteristic bands selected by the band selection module in the network Y–Net proposed in this paper. “The importance score” is determined by the weight of the one–dimensional convolution kernel in the band selection module. The weight of the convolution kernel in the one–dimensional convolution layer represents the band’s importance. The greater the absolute value of the weight, the more important the corresponding band is. After network training is completed, the weight of each convolution kernel is a fixed constant, representing “the importance score” of each band. It can be noticed that with the constraint of adding the sum of weights to the loss function, the weights of some bands are adjusted to be close to zero. The bands with importance scores between –1 and 1 were removed, and the remaining bands were used as characteristic bands. The black curve in Figure 6b is the spectral curve of corn anthracnose, and the pink area is the region where the characteristic bands selected by the band selection module are located. It can be found that they are mainly concentrated in the five regions of 409 nm, 415 nm, 501–596 nm, 635–732 nm, 815–916 nm, and 978–996 nm, which are all in the three regions of visible light, red edge, and near–infrared. Figure 6c shows the regression coefficient of each band calculated by the partial least squares regression algorithm, which is between 0 and 1. “The importance score” represents the regression coefficient, and the greater the absolute value of the regression coefficient, the greater the importance of corresponding bands. The characteristic bands are mainly concentrated in the 507–552 nm and 611–688 nm regions in the near–infrared and red–edge regions. Figure 6d shows the bands selected by the continuous projection algorithm. The green curve is the spectral curve of corn anthracnose, and the red area is the characteristic band selected by the continuous projection algorithm. In order to be consistent with the method in this paper, a total of 43 feature bands were selected, mainly concentrated in the 786–901 nm and 993–999 nm regions in the near–infrared region.

According to the description in Section 3.1, it can be found that the feature bands selected by the above three methods are mainly concentrated in the region with apparent changes in spectral characteristics. Furthermore, through PLS and SPA selected out of the feature band, most are concentrated in the band selection module selected out of the feature band region, which also further explains the effectiveness of the band selection module.

### 3.4. Analysis and Comparison of Classification Results

#### 3.4.1. Comparison of Y–Net and Traditional Methods

**Figure 7:**
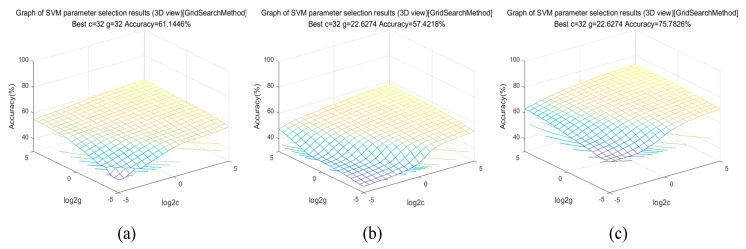
Grid search results for the support vector machine (SVM) model. (**a**) Optimal parameter search results for the PLS–SVM model; (**b**) optimal parameter search results for the SPA–SVM model; (**c**) optimal parameter search results for the Y–Net(band)–SVM model.

This paper uses two representative band selection methods (PLS and SPA) to compare with the feature bands extracted by Y–Net to verify the effectiveness of the Y–Net proposed in this study. In addition, SVMs with radial basis functions [34] were used for comparison. The search range was set to –32–32, and the grid search results are shown in Figure 7. The optimal parameters of the optimized PLS–SVM model were c = 32 and g = 32. The optimal parameters of the SPA–SVM model were c = 32 and g = 22.6274. The Y–Net(band)–SVM model’s optimal parameters were c = 32 and g = 22.6274.

To match the number of bands extracted by the band selection module, 43 characteristic bands were selected by each PLS and SPA for experimental comparison with the characteristic bands extracted by the band selection module. Table 1 summarizes the classification accuracy of SVM and Y–Net under different feature extraction algorithms. As seen from the first three rows of Table 1, the classification accuracy of the SVM ranges from 0.5742–0.7578. The classification accuracy of SVM was 0.6114 and 0.5742 after inputting PLS and SPA–extracted feature bands into SVM, respectively. The accuracy of the Y–Net(band) selected from Y–Net reached 0.7578 after inputting it into SVM. The experimental analysis results show that SPA has limited performance improvement compared to the two traditional feature extraction methods compared to the PLS method. The main reason is that the feature selection process of the method is unsupervised, and the variables selected maximize the explanation of the independent variable space without building a predictive model, so the explanatory ability of variables is limited. The band selection module of Y–Net is mainly based on a deep convolutional neural network, which retains not only spectral features but also learns spatial features. Therefore, these bands can obtain the highest classification accuracy after the input to SVM, which are much higher than the previous two traditional feature extraction methods.

In this study, to independently verify the classification performance of Y–Net, the band selection module of Y–Net is removed, and only the attention module, 3D–2D hybrid CNN module, and classification module were left for training, which was named Y–Net(w). After inputting the PLS and SPA extracted feature bands into Y–Net(w), the classification accuracy of Y–Net(w) was 0.6475 and 0.5562, respectively, and the classification accuracy after inputting the Y–Net selected band Y–Net(band) into Y–Net(w) was slightly improved compared to the traditional feature extraction method, reaching 0.9653. Experimental analysis showed that the classification accuracy of all two traditional feature extraction methods in Y–Net was significantly improved compared to using the conventional classifier SVM, and the highest classification accuracy of 0.9653 was achieved when using the features extracted from the band selection module as input, which also further illustrates the effectiveness of the method in this study. Overall, the classification performance of Y–Net(w) is better than that of SVM methods, and the classification results of Y–Net(band) features are better than those of PLS and SPA methods. Therefore, both the model and the model’s feature selection method are crucial.

Comparing rows 6 and 7 of Table 1, it can be seen that Y–Net achieves an accuracy of 0.9737 when using the original hyperspectral image as input. Compared with only using 43 feature bands extracted, the accuracy is improved by 0.0084. It shows that the full band selection contains more information and has better classification accuracy. The experiments show the superiority of the Y–Net proposed in this study.

#### 3.4.2. Comparison of Y–Net and Other Networks

To verify the superiority of the 3D–2D hybrid CNN, the 43 feature bands (Y–Net(band)) extracted from the band selection mode of Y–Net were fed into CNN–ATT [25] and PCNN [24] for comparison with Y–Net(w) in this study.

CNN–ATT used a one–dimensional convolutional neural network, so the 43 feature bands of each hyperspectral image sample were selected before input and compressed into a 1 × 43 vector as input. PCNN used a two–dimensional convolutional neural network, so there was no need to process hyperspectral images, and only 43 characteristic bands could be taken as input. 

**Figure 8:**
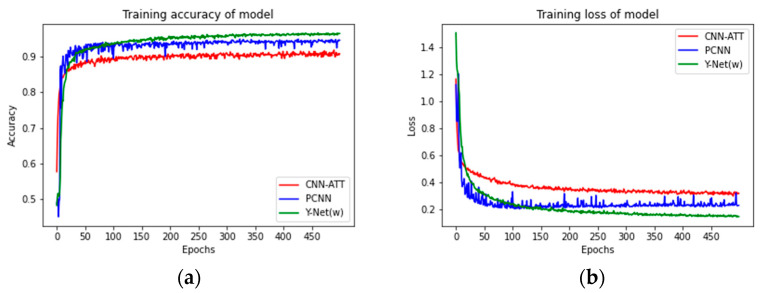
(**a**) Comparison plots of training accuracy and the number of iterations; (**b**) comparison plots of loss values and the number of iterations.

Figure 8 shows each model’s variation in accuracy and loss function values. As can be seen from the graph, the accuracy increases rapidly, and the loss values decrease rapidly in the first few epochs of each model. As the network continues to be trained, the network gradually stabilizes. As can be seen from Figure 8, the Y–Net(w) model is more accurate than the PCNN and CNN–ATT models. The specific accuracy rates are shown in Table 2. The model of CNN–ATT achieved a classification performance of 0.9081. The model of PCNN achieved a classification performance of 0.9444. The Y–Net(w) model achieved an accuracy of 0.9653, which was 0.0572 and 0.0209 higher than the CNN–ATT model and the PCNN model. The results show that the Y–Net(band)–(Y–Net(w)) achieved better classification performance compared with 1D–CNN and 2D–CNN. The superiority of the 3D–2D hybrid CNN used in this study was verified.

### 3.5. The Impact of the Band Selection Module on Classification

**Figure 9:**
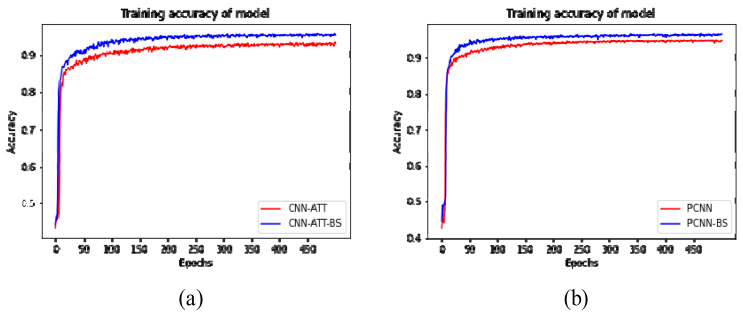
Accuracy comparison before and after the addition of the band selection module. (**a**) Accuracy of CNN–ATT before and after adding the band selection module; (**b**) accuracy of PCNN before and after adding the band selection module.

This experiment verifies the performance of applying the band selection module in PCNN and CNN–ATT. For each group, we used the raw HSI data to train CNN models with and without the band selection module. CNN with band selection modules is called PCNN–BS and CNN–ATT–BS. Figure 9 shows the difference in accuracy with and without the band selection module. PCNN and CNN–ATT have significantly improved their accuracy by including the band selection module. The accuracy of PCNN improved from 0.9482 to 0. 9654. The quasi–accuracy of CNN–ATT improved from 0.9342 to 0.9541. Therefore, the band selection module proposed in this paper can help build a higher–quality model. Moreover, adding the band selection module to the CNN model does not increase the processing time either. The band selection module enhances the operational capability of the CNN model (it not only learns how to classify HSI pixels efficiently but also selects the influential HSI bands). 

### 3.6. Lightweight Y–Net Based on Network Pruning

Although deep learning methods can acquire in–depth features of hyperspectral images, using all the band information of a hyperspectral image as input can result in a trained model that takes up too much memory. To solve this problem, the traditional approach is first to dimension reduction of the hyperspectral images, such as PCA, and then use the downscaled hyperspectral image data to train a deep learning model. However, these methods are independent of the model’s training, so they are difficult to achieve global optimality, and the accuracy of traditional dimensionality reduction methods is unsatisfactory through comparative experiments. So, when using raw hyperspectral data as input, it is necessary to solve the problem of oversized models and ensure that there is no loss of model accuracy.

**Figure 10:**
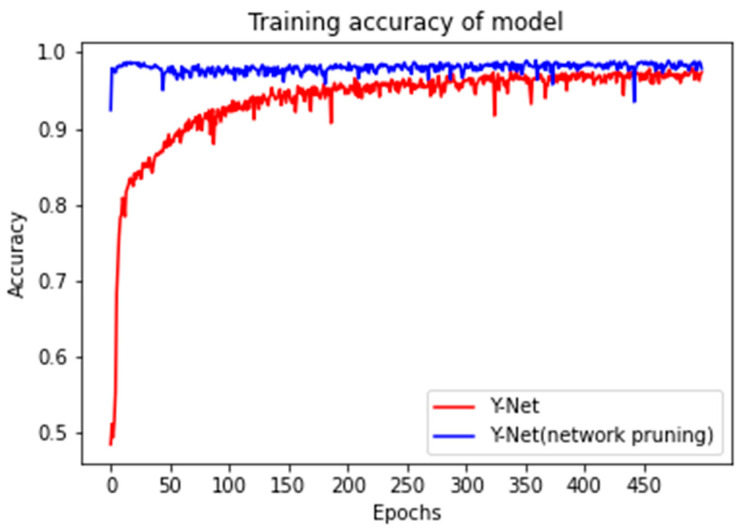
Accuracy of Y–Net before and after pruning.

Network pruning removes certain unimportant parts of a CNN while maintaining the performance. Depending on the object of deletion, pruning can be divided into weight pruning, which removes unimportant weights in the CNN, and neuron pruning, which removes unimportant neurons in the CNN [35]. This study focuses more on the magnitude of the weights, so weight pruning was used. The results after pruning are shown in Table 3 and Figure 10. Figure 10 shows that before pruning, the network was trained using the full band and achieved an accuracy of around 97.37. When pruned, the accuracy of the network was basically stable at around 98.34. It can be seen that the network pruning removes redundant parameters that do not contribute significantly to the accuracy of the results. Not only does it not affect the accuracy of the model, but it also improves it slightly. In the band selection module, bands may be pruned when the weights are zero or close to zero so that those bands that are not significant are removed. Thus, the computational complexity of the subsequent modules is reduced, and the pruned network is retrained to escape the probability of the previous local minimum and further improve accuracy. Table 3 shows the Y–Net before and after network pruning, which reduces the model size to 1/3 of the original size and improves the model accuracy by 0.0097. 

The network pruning does not require the bands selected by the Y–Net band selection module to be re–entered into the model for training and significantly reduces the model’s size while ensuring classification accuracy. Moreover, a large, trained network is reduced to a smaller one, enabling neural networks to be deployed in resource–constrained environments. In contrast to the two studies in [24,25], the authors used the network model for feature band selection and then retrained the model with the selected bands, and both the retrained models have reduced classification accuracy compared to the full–band models. So, it is good to train directly with the full band and then perform network pruning.

### 3.7. Effectiveness of Y–Net for Disease Identification in Other Crops

**Figure 11:**
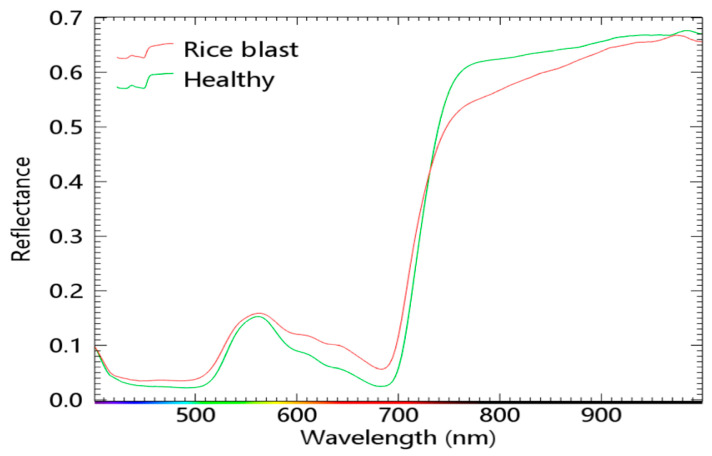
Mean spectral profiles of healthy and diseased rice leaves.

**Figure 12:**
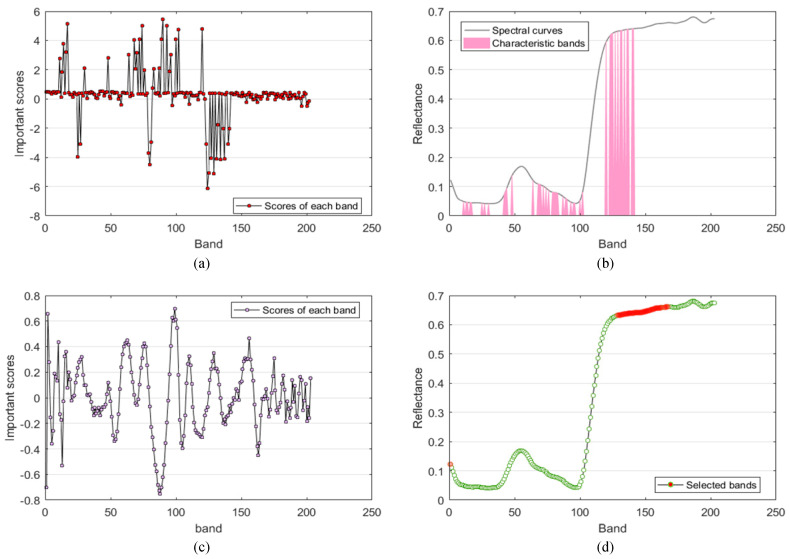
Results of feature band selection. (**a**) The importance score of each feature channel calculated by the Y–Net band selection module. (**b**) The region of the spectral curve where the feature band extracted by the band selection module is located. (**c**) The magnitude of the regression coefficients of each band by PLS calculations. (**d**) Feature bands by SPA selection.

**Figure 13:**
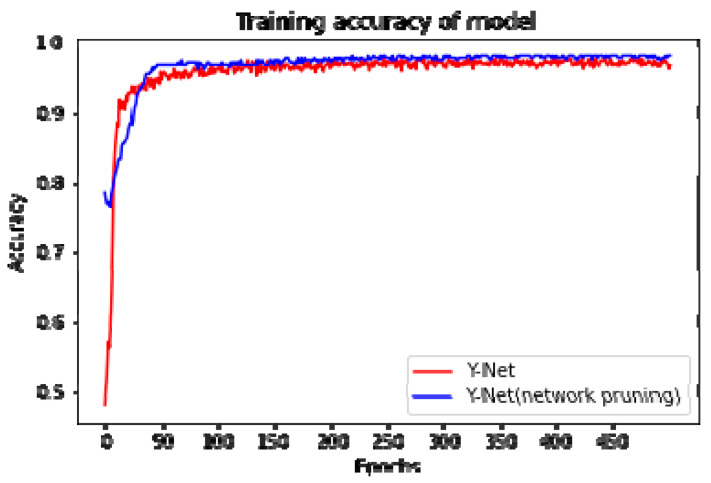
Accuracy of Y–Net before and after pruning.

To further validate the effectiveness of the model proposed in this paper, we conducted experiments using hyperspectral data from healthy and diseased rice leaves, with a total of 2272 regions of interest (1152 healthy and 1120 diseased). These data were obtained from [36]. Figure 11 shows the average spectral profiles of healthy and diseased rice leaves. Figure 12 shows the results of the feature band selection. Figure 12a shows that the band selection module can extract the feature bands when applied to other data sets. Figure 12b shows that the selected characteristic bands are mainly concentrated in 430–540 nm, 587–700 nm, and 753–815 nm. Comparison with Figure 11 shows that these characteristic bands are contained in regions where the spectral profiles of healthy and diseased leaves are significantly different. Figure 12c,d also shows that the feature bands selected by PLS and SPA are also concentrated in these regions and included in the feature band regions selected by the band selection module. Figure 13 compares the accuracy before and after Y–Net pruning using full band training. Before pruning, the accuracy reached about 0.9677. After pruning, the network’s accuracy was stable at around 0.9709.

From the experimental results, the Y–Net proposed in this paper has good results in hyperspectral image classification and feature extraction. The model can classify other crop diseases by improving the Y–Net model or adjusting the model parameters.

### 3.8. Advantages and Disadvantages of Y–Net

The results of the above comparison experiments all illustrate the capability of the Y–Net proposed in this paper. We not only compare Y–Net with traditional feature selection methods, traditional classifiers, and other CNNs but also investigate the impact of applying the band selection module in other CNNs. The experimental results all show that Y–Net has a good classification effect and that the band selection module can effectively select the important bands of HSI. The band selection module does not affect the training time or the classification accuracy of the underlying model. The band selection module embedded in the CNN model proposed in this paper can compete with traditional feature selection methods. Subsequently, we conducted experiments on the network model proposed in this paper using another dataset, rice, and could find that good results were also achieved.

Our experiments provide quantitative, qualitative, and statistical evidence on the capabilities of the proposed Y–Net and allow us to gain insight into our technique’s most important strengths and weaknesses—they have been summarized in Table 4.

## 4. Conclusions

This study proposes a Y–Net model to identify corn diseases in combination with hyperspectral images. The method integrates feature selection, extraction, and classification into a single system, enabling end–to–end identification of corn diseases. The feature bands extracted by the Y–Net band selection module are compared with the two conventional feature selections, PLS and SPA. Comparative experiments subsequently confirm the advantages of the bands extracted by the Y–Net band selection module. Finally, the trained Y–Net was pruned. The pruned Y–Net not only slightly improved the classification accuracy but also reduced the model size to one–third of the original size, significantly reducing the memory requirement of the computer. Compared with traditional methods, Y–Net considers both spatial and spectral features of hyperspectral images in feature selection, feature extraction, and classification, making the selected bands more representative and robust. The overall results show that deep learning combined with hyperspectral images has excellent potential in feature selecting and classifying corn diseases. In addition, future studies should use more varieties of crop disease samples to test the robustness of the proposed network.

## Figures and Tables

**Table 1:** Results of the classification of corn diseases.

Model	Number of Features	Training Set (%)	Test Set (%)
PLS–SVM	43	62.39	61.14
SPA–SVM	43	59.11	57.42
Y–Net(band)–SVM	43	76.52	75.78
PLS–(Y–Net(w))	43	65.26	64.75
SPA–(Y–Net(w))	43	55.88	55.62
Y–Net(band)–(Y–Net(w))	43	97.62	96.53
Y–Net	203	98.57	97.37

**Table 2:** Classification performance of Y–Net(w) and other networks.

Model	Training Set (%)	Test Set (%)
Y–Net(band)–Y–Net(w)	97.68	96.53
Y–Net(band)–(CNN–ATT)	86.79	85.37
Y–Net(band)–PCNN	96.66	94.04

**Table 3:** The results of network pruning.

Model	Model Size	Test Set (%)
Y–Net (network pruning)	8.92 MB	98.34
Y–Net	25.31 MB	97.37

**Table 4:** Advantages and disadvantages of Y–Net.

Advantages	Disadvantages
The selected characteristic bands are well representative	The number of characteristic bands selected needs to be chosen manually
The band selection module does not affect the training time and classification performance of the model	For small sample datasets, if the network does not converge well, it will be challenging to select a specific number of feature bands in the band selection module when the weights of the convolution kernels of the 1D convolution are challenging to converge to 0
3D–2D hybrid models can extract better spectral and spatial features of HSI than 2D–CNN and 1D–CNN	
The band selection module can be seamlessly adapted to convolutional neural network architectures	

## Data Availability

Raw data are available from the authors upon request.
